# A Biocompatible ZIF-8 Spray and Its Long-Lasting Antibiosis

**DOI:** 10.3390/nano16110672

**Published:** 2026-05-27

**Authors:** Jiaxi Xia, Xiaojing Zhang, Dayan Ma, Chunmei Tang, Xia Lou, Wei Wang, Lan Zhang

**Affiliations:** 1 School of Apparel and Art Design, Xi’an Polytechnic University, Xi’an 710048, China; 42306310123@stu.xpu.edu.cn; 2 State Key Laboratory for Mechanical Behavior of Materials, Xi’an Jiaotong University, Xi’an 710049, China; xiaojing.zhang0216@gmail.com (X.Z.); madayan@mail.xjtu.edu.cn (D.M.); 3 State Key Laboratory of Clean and Efficient Turbomachinery Power Equipment, Deyang 618000, China; tangcm0040@dongfang.com (C.T.); louxia@dongfang.com (X.L.); 4 Dongfang Electric Corporation Dongfang Turbine Co., Ltd., Deyang 618000, China

**Keywords:** antibacterial spray, ZIF-8, biocompatible, long-lasting

## Abstract

Current antibacterial sprays face major limitations, including rapid evaporation, short-lived efficacy, skin irritation, and poor adhesion to surfaces, highlighting an urgent need for a durable and biocompatible alternative. To address these challenges, we developed a ZIF-8-based spray (ZNS-WO20) composed of ZIF-8 nanoparticles dispersed in 50% ethanol and 20% OTES. OTES acts as a dispersant and binder, enabling wash-resistant coatings on gauze and glass. ZIF-8 exhibits pH-responsive Zn^2+^ release, achieving nearly 100% killing of *S. aureus*, *E. coli*, and methicillin-resistant *S. aureus* (MRSA) at 160 μg/mL through intracellular reactive oxygen species (ROS) generation. The spray maintains >95% antibacterial efficacy against *S. aureus* after five washing cycles and seven days of outdoor exposure, and causes no dermal irritation in rats. This work fills the gap for a long-lasting, skin-friendly antibacterial spray, showing promise for healthcare disinfection and surface protection.

## 1. Introduction

COVID-19 ravaged the globe during 2019–2021. Although many strategies were applied to kill the virus and inhibit its transmission, the lives of more than 7 million people were threatened. Its main method of spread is contacting transmission. Various methods were adopted to kill the virus on object surfaces or skin during the pandemic, including UV irradiation, boiling and spraying disinfectors, according to different situations; such strategies are similar to antibacterial strategies. Among them, sprays (e.g., 75% ethanol and diluted benzalkonium chloride) are relatively convenient for use on the human body and objects with complicated shapes. According to the dispersing solution, sprays mainly have three types: aqueous-based, organic solution-based and mixed types [1,2]. They each have their own advantages and drawbacks. For example, organic solvents like ethanol or propylene glycol can solve lipophilic antimicrobial agents, forming homogeneous solutions, such as medical disinfectant sprays containing 75% ethanol dissolved with chlorhexidine [3]. They can kill the adhering bacteria effectively and immediately; however, their short duration leads to frequent spraying requirements, and irritation can trouble the users. Water-based sprays are generally more compatible and milder than organic-based sprays; for example, pure water with polyhexamethylene biguanide (PHMB) can be used for infants and skin wound disinfection [4]. Unfortunately, sprays are limited by the dispersibility or solubility of antimicrobial agents in water. Emulsion sprays that mix oil and water as solvents enable the simultaneous delivery of hydrophilic and oleophilic antimicrobial agents; however, they have complicated components [5]. So, a new spray system is needed to address the above problems and achieve appropriate antibacterial duration, stability and biocompatibility simultaneously.

The virus/bacteria elimination efficiencies depend on the types and concentrations of antibacterial agents. Antibacterial agents can be broadly classified into three categories: organic disinfectants (e.g., alcohols, quaternary ammonium compounds, chlorhexidine), inorganic antimicrobials (e.g., metal ions, metal oxides, metal–organic frameworks), and natural antimicrobials (e.g., chitosan, antimicrobial peptides). Organic types generally act rapidly via protein denaturation or membrane disruption, offering low cost and immediate efficacy. However, they are volatile, short-lived, require frequent reapplication, and may cause skin irritation. Quaternary ammonium compounds have longer residual activity but pose a risk of promoting bacterial resistance [6]. Natural antimicrobials are relatively expensive, easily inactivated, and require low-temperature storage [7]. Inorganic agents (e.g., Ag^+^, Cu^2+^, Zn^2+^, ZnO, AgNPs) provide broad-spectrum activity with low resistance risk. Thereinto, metal ions (e.g., Ag^+^, Cu^2+^ and Zn^2+^) have unique advantages in antibiosis, such as various forms and controllability [8]. In particular, zinc is an essential trace element for the human body. Compared with Ag^+^ and Cu^2+^ ions, Zn^2+^ has a relatively high biocompatible threshold and is widely studied in antibiosis [8,9,10,11,12]. Many kinds of zinc compounds have been used as antibacterial agents, such as ZnO, Zn_3_PO_4_, and ZnSO_4_ [9,13]. Among them, zeolitic imidazolate framework-8 (ZIF-8) is a subclass of metal–organic frameworks with Zn^2+^ ions as metal nodes connected by 2-methylimidazolate organic linkers. ZIF-8 shows outstanding antibacterial and antivirus properties due to the synergistic effect of Zn^2+^ and the five-membered heterocyclic structure of imidazole [11,12,13,14]. In particular, the imidazolate framework contains acid-unstable organic ligands, which can release Zn^2+^ in an acidic microenvironment to kill bacteria responsively. Nevertheless, ZIF-8 shows concentration-dependent antibacterial activity and biocompatibility with opposing trends: higher concentrations enhance antibiosis but reduce biocompatibility [11]. Various surface modification strategies have been employed to endow ZIF-8 with multi-biofunctions. For instance, hyaluronic acid (HA), bovine serum albumin (BSA), polydopamine, and maltodextrin have been widely used to enhance the stability and cytocompatibility of ZIF-8 while preserving its pH-responsive antibacterial activity [11,15]. However, ZIF-8 is mainly applied as the powder form, which cannot adhere well on the surface subjects, limiting the application in antibacterial sprays. Surfactants and dispersants such as polyurethane (PU)–polymethyl methacrylate (PMMA), poly(vinyl alcohol) (PVA), and polydimethylsiloxane (PDMS) have been incorporated into ZIF-8-based sprays to improve the uniform distribution and adhesion of ZIF-8, as well as to provide durable resistance against viral and bacterial pathogens [16,17,18]. Unfortunately, most of these systems either require complex multi-step modifications, rely on non-biocompatible organic solvents, or lack systematic evaluation of long-term durability and biosafety.

Silane couplers can chemically bond with hydroxyl groups, transforming surfaces from hydrophilic to hydrophobic and thereby improving nanoparticle dispersibility and waterproofing properties, which have been wildly applied in surface modification. Among them, N-octyltriethoxysilane (OTES) is economical and can form stable siloxane bonds on inorganic/organic material surfaces, thereby improving material adhesion and dispersion [19,20]. However, whether it can be applied in a ZIF-8-containing spray to increase comprehensive performance is unknown. In this work, a spay consisting of ethanol, water, OTES and ZIF-8 nanoparticles (NPs) was proposed. ZIF-8 NPs were fabricated via the solvothermal method and then dispersed in an ethanol–water–OTES mixed solution to form the spray. ZIF-8 in spray form killed bacteria effectively, and OTES endowed the spray with antibacterial durability and water resistance. The microstructure of ZIF-8 NPs and their antibacterial properties in spray form were examined, and an ethanol–water–OTES mixed biocompatible ZIF-8 spray which can form durable antibacterial films on different objects was obtained. This work proposes a practical spray to achieve long-lasting bacterial resistance for different objects, and it can potentially be applied to prevent the spreading of viruses and bacteria in the future.

## 2. Experimental Methods

### 2.1. Sample Preparation

**Materials and reagents:** Zinc nitrate hexahydrate (Zn(NO_3_)_2_·6H_2_O, 99.99%, reagent grade, Aladdin Industrial Corporation, Shanghai, China) and 2-methylimidazole (C_4_H_6_N_2_, 99%, reagent grade) were purchased from Sigma-Aldrich Corporation, USA. Methanol (CH_3_OH, ≥99.8%, HPLC grade) was obtained from Sinopharm Chemical Reagent Co., Ltd., Shanghai, China. N-Octyltriethoxysilane (OTES, C_14_H_32_O_3_Si, 98%, technical grade) was supplied by Aladdin Industrial Corporation, Shanghai, China. All chemicals were used as received without further purification.

**Synthesis of ZIF-8 nanoparticles:** The synthesis was carried out under ambient atmospheric pressure and at room temperature. A total of 0.25 mol of Zn(NO_3_)_2_·6H_2_O was dissolved in 30 mL of methanol under magnetic stirring at 300 rpm for 10 min to form a clear solution (Solution A). A total of 1 mol of 2-methylimidazole was dissolved in 30 mL of methanol under the same stirring conditions to obtain Solution B. Subsequently, Solution B was poured quickly into Solution A, and the mixture was immediately subjected to ultrasound treatment (40 kHz, 200 W) using an ultrasonic cleaner (KQ-300DE, Kunshan, China) for 30 min. Then, the suspension was centrifuged at 10,000 rpm (12,000× *g*) for 10 min. The supernatant was discarded, and the precipitate was washed three times alternately with 30 mL of absolute ethanol and 30 mL of deionized water to remove unreacted reagents. The final product was collected and dried in a vacuum oven (DZF-6020, Yiheng, Shanghai, China) at 60 °C for 12 h. The dried ZIF-8 NPs were stored in a desiccator at room temperature for further characterization and spray formulation.

**Preparation of ZIF-8-based spray:** The as-synthesized ZIF-8 NPs were dispersed in ethanol/water/OTES mixed solvents to achieve a final ZIF-8 concentration of 1 mg/mL. Different sprays were prepared as follows: (i) ZNS-E: absolute ethanol only; (ii) ZNS-W25 and ZNS-W50: ethanol/water mixtures containing 25 vol% or 50 vol% water, respectively (no OTES); (iii) ZNS-WO5, ZNS-WO10, and ZNS-WO20: 50 vol% ethanol/50 vol% water containing 5 vol%, 10 vol%, or 20 vol% OTES, respectively. Each mixture was shaken thoroughly using a vortex oscillator (MS3 basic, IKA-Werke GmbH & Co. KG, Germany) for 10 min to ensure homogeneous dispersion. The resulting spray suspensions were transferred into commercial spray bottles (nozzle diameter 0.3 mm, volume 30 mL) and stored at room temperature. Each spray actuation delivered approximately 160 μL of solution, calibrated by weighing.

### 2.2. Material Characterization

A field emission scanning electron microscope (FESEM; SU6600, Hitachi Ltd., Japan) and transmission electron microscope (TEM; JEM-2000FX, JEOL Ltd., Japan) were used to observe the morphology and detect the element composition of the nanoparticles. Dynamic light scattering (DLS; Zetasizer Nano ZSE, Malvern, UK) was applied to analyze nanoparticle hydrodynamic diameter. X-ray diffraction (X’Pert PRO, PANalytical B.V., The Netherlands) was used to detect the crystalline structure of nanoparticles. The pH-responsive release of Zn^2+^ ions from ZIF-8 NPs was evaluated by immersing 10 mg of ZIF-8 powder in 10 mL of phosphate-buffered saline (PBS) at different pH values (7.4, 6.5, and 5.5) for 24 h. The concentration of released Zn^2+^ ions was quantified using inductively coupled plasma optical emission spectrometry (ICP-OES, PerkinElmer Optima 8000, PerkinElmer, Inc., USA). Each experiment was performed in triplicate, and the cumulative Zn^2+^ release was calculated and expressed as the mean ± standard deviation (ppm).

### 2.3. In Vitro Antibacterial Test

The antibacterial behaviors of samples were evaluated against *Staphylococcus aureus* (*S. aureus*, ATCC 25923), *Escherichia coli* (*E. coli*, ATCC 8739) and methicillin-resistant *staphylococcus aureus* (MRSA, ATCC 33591). Bacteria were incubated in beef extract-peptone medium (BEP, Qingdao Hi-Tech Industrial Park Hope Bio-Technology Co., Ltd., Qingdao, China) at 37 °C for 12 h, then adjusted to 10^5^ CFU/ mL in nutrient broth. A total of 500 mL of the samples were placed in 24-well plates, added with 500 mL bacterial suspension and incubated at 37 °C. After incubation for different durations, the bacterial culture medium was taken out and diluted on an agar plate to evaluate the antibacterial activity using the plate-counting method. The antibacterial ratio was obtained according to the formula Ra (%) = (C_0_ − C_t_)/C_0_ × 100%, where C_0_ and C_t_ were the numbers of live bacteria of control and test groups, respectively.

The antibacterial efficiencies of sprays were also evaluated using the agar disc diffusion and zone of inhibition method. Sterile commercial gauze slices (approximately 6 mm in diameter) were used as the substrate. The slices were placed in a sterile Petri dish. A total of 160 µL of the test spray solution was applied directly onto each sterile slice using a spray bottle to ensure the entire slice was evenly wetted. The treated slices were then left to dry aseptically in a laminar flow cabinet at room temperature for 30 min. The adjusted bacterial inoculum was evenly spread onto the surface of sterile agar plates using a sterile cotton swab to create a confluent lawn of bacteria. Subsequently, the dried slices were gently placed onto the center of the inoculated agar plates using sterile forceps. All plates were incubated at 37 °C for 18 h. Following incubation, the diameters of the clear zones of inhibition were measured in millimeters using a digital caliper. Each test and control were performed in triplicate to ensure reproducibility. The results were expressed as the mean zone of inhibition (in mm) ± standard deviation.

Bacteria on the sprayed gauze slices after incubation for different times were also examined by using the plate-counting method. Bacteria were further stained with the Live/Dead@Baclight™ Bacterial Viability Kit (L13152, Thermo Fisher Scientific Inc., USA) according to the operating manual. Fluorescence images were obtained by using a fluorescence microscope (SMZ745T, Nikon Co., Japan). For FESEM observation, bacteria were collected, fixed with 2.5% glutaraldehyde, dehydrated in gradient alcohol ethanol, then vacuum-dried and sputtered with gold. Intracellular ROS was assessed using DCFH-DA probes (Beyotime Biotech Inc., Shanghai, China) according to the instruction and observed by using a fluorescence microscope.

### 2.4. In Vitro Biocompatibility

#### 2.4.1. Cell Culture

The mouse fibroblast cell line (L929) was purchased from the Stem Cell Bank (Shanghai, Chinese Academy of Sciences) and cultured in a humidified atmosphere incubator with 5% CO_2_ at 37 °C. Cells were cultured in alpha minimal essential medium (α-MEM, Thermo Fisher Scientific Inc., USA) supplemented with 10% (*v*/*v*) Fetal Bovine Serum (FBS, Thermo Fisher Scientific Inc., USA), 1 mM sodium pyruvate and 15 mM NaHCO_3_. The cultures were maintained at 37 °C in a humidified atmosphere containing 5% CO_2_. Upon reaching 80–90% confluence, the cells were detached using 0.25% trypsin-EDTA (Thermo Fisher Scientific Inc., USA) for subculturing and subsequent experiment. The complete medium was replaced every 2 days.

L929 cells were seeded in culture medium with different concentrations of ZIF-8 or medium extracts of gauze samples at a density of 1 × 10^4^ cells/well. After varying hours of incubation, the mitochondrial activity of cells was assessed by using the MTT (3-(4,5-dimethylthiazol-2-yl)-2,5-diphenyltetrazolium bromide) colorimetric assay according to the instructions. For the extract preparation, the sprayed gauze samples were immersed in medium with a surface area-to-volume ratio of 3 cm^2^/mL according to ISO 10993-12 [21], and then incubated at 37 °C for 24 h with gentle agitation. After incubation, the mixture was centrifuged and the supernatant was filtered through a 0.22 μm sterile filter to obtain the stock extract (100%). Serial dilutions were prepared with fresh culture medium for dose–response assays.

#### 2.4.2. Evaluation of Dermal Irritation

The acute dermal irritation/corrosion potential of the antibacterial spray was evaluated. Twenty male Sprague-Dawley rats about 250 g in weight were anesthetized by intraperitoneal injection of 2.5 Wt% pentobarbital sodium solution. Approximately 24 h before the test, the fur on the dorsal area of the animals was removed by clipping 4 h prior to application. Only animals with healthy, intact skin were used. Three animals were used for each group. The test substance was applied to a small area (approximately 6 cm^2^) of intact skin on one side of each animal. The site was covered with a gauze patch and held in place for a 0–60 min exposure period. Every 30 min, gauzes were sprayed with about 0.5 mL spray solution. Sterile physiological saline solution (PBS) was served as a negative control. Complete benzalkonium bromide and 10 vol% benzalkonium bromide solution were applied as positive controls. Benzalkonium bromide is known to be a mild dermal irritant and served as a benchmark for comparison. After the different exposure durations, the gauzes were removed, and any residual test substance was gently wiped away without disturbing the integrity of the skin. The skin was observed for signs of erythema and edema at removal. The reactions were scored according to the Draize skin scoring system (Table 1). All animal procedures were approved by the Xi’an Jiaotong University Laboratory Animal Ethics Committee with Approval No. XJTUAE2024-1792 (Approval date: 11 March 2024). Statistical analysis was performed using SPSS 16.0, with significance thresholds set at * *p* < 0.05 and ** *p* < 0.01.

## 3. Results and Discussion

### 3.1. The Microstructure and Properties of ZIF-8 NPs

**Figure 1:**
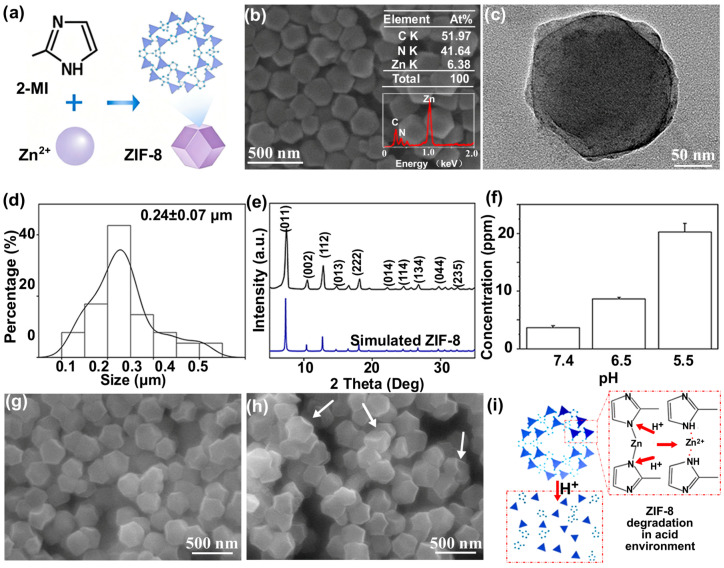
(**a**) A schematic illustration of the synthesis of ZIF-8 NPs via coordination between Zn^2+^ and 2-methylimidazole linkers, (**b**) a SEM image of ZIF-8 NPs, insets showing the corresponding EDAX spectra and the quantified element contents, (**c**) a TEM image of ZIF-8 NPs, (**d**) the XRD pattern of ZIF-8 NPs, (**e**) hydrodynamic diameter distribution of NPs by DLS, (**f**) cumulative Zn^2+^ release from ZIF-8 nanoparticles in PBS at pH 7.4 (neutral), pH 6.5 (acidulous) and pH 5.5 (acidic) after 1 day of immersion, SEM images of ZIF-8 after 1 day of immersion in neutral solution (**g**) and in acidic solution (**h**), and (**i**) schematic diagram of the pH-responsive degradation mechanism of ZIF-8.

ZIF-8 formation relies on coordination interactions between Zn^2+^ ions and 2-methylimidazolate linkers. Each Zn^2+^ ion tetrahedrally coordinates with four nitrogen atoms from four distinct linkers, forming tetrahedral building blocks. These blocks aggregate into amorphous precursor particles and undergo an amorphous-to-crystalline transition to yield the ordered framework, ultimately forming mature ZIF-8 crystallites [22], as illustrated in Figure 1a. The nanoparticles are rich in Zn, C and N, and have polyhedral morphologies with an average diameter of 245 nm (Figure 1b,c), which is further confirmed by the dynamic light scattering analysis as shown in Figure 1d. X-ray diffraction was employed to confirm the phase composition of nanoparticles (Figure 1e). The diffraction peaks match well with the simulated ZIF-8 pattern, and no peaks corresponding to other materials were detected [23]. For instance, the peaks at 10.4°, 12.7°, and 18.0° correspond to the (002), (112), and (222) planes of ZIF-8, respectively. These results demonstrate the successful preparation and high purity of ZIF-8 nanoparticles.

Zn^2+^ exhibits dose-dependent antibacterial activity and cytotoxicity [9,11]. Zn^2+^ at a concentration of 4 ppm has been demonstrated to kill 97.22% of *S. aureus* [24], and its release kinetics depend on the degradation rate of ZIF-8, which is related to the pH of the microenvironment [11,25]. Many bacterial species are capable of metabolizing available carbohydrates through glycolytic pathways. A primary end-product of this anaerobic fermentation is organic acids, including lactic acid, acetic acid, and formic acid, and these acidic metabolites lead to an acidic microenvironment with a pH value of 4.5–6.5, which can accelerate ZIF-8 degradation to kill bacteria [26]. Therefore, Zn^2+^ release from nanoparticles was evaluated in a neutral microenvironment (pH = 7.4) and simulated infected microenvironments with pH = 6.5 and pH = 5.5, respectively. After 1 day of immersion, 3.7 ppm of Zn^2+^ was detected in the phosphate-buffered system (PBS) solution with a pH of 7.4 (Figure 1f), which is within the biocompatible range [9,11]. Meanwhile, under acidic conditions, Zn^2+^ release was significantly accelerated, reaching 8.7 ppm at pH 6.5 and 20.3 ppm at pH 5.5 (Figure 1f), indicating accelerated degradation of ZIF-8 in environments with lower pH values. The Zn^2+^ concentrations in PBS at pH 6.5 and pH 5.5 are much higher than the minimum bactericidal concentration (MBC) of Zn^2+^, and should kill bacteria effectively [9,18,27]. The acid-responsive degradation of ZIF-8 is further confirmed by SEM observation of nanoparticles after immersion. As shown in Figure 1g,h, compared with the as-prepared ZIF-8, slightly changes in size and morphology are observed after immersion in neutral solution (Figure 1g), indicating the chemical stability of ZIF-8 in pure water or ethanol; however, many dimples are observed on ZIF-8 NPs after immersion in acid solution (marked as arrows in Figure 1h). This accelerated release arises from the protonation of 2-methylimidazole ligands in ZIF-8 under acidic conditions [28], which disrupts coordination bonds between Zn^2+^ and 2-methylimidazole, inducing Zn^2+^ releasing, as illustrated in Figure 1i.

**Figure 2:**
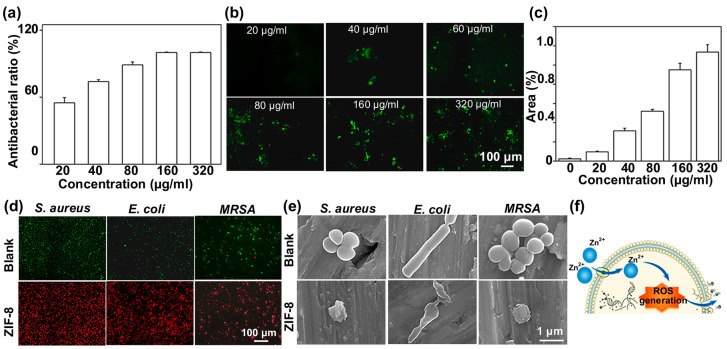
(**a**) Antibacterial activity of ZIF-8 nanoparticles against *S. aureus* at different concentrations, (**b**) fluorescence images of intracellular ROS levels in *S. aureus*, (**c**) quantitative analysis of relative ROS fluorescence intensity corresponding to (**b**), (**d**) Live/Dead staining of bacteria after 24 h incubation without (Blank) and with 160 μg/mL ZIF-8, (**e**) SEM images of *S. aureus* morphology, and (**f**) a schematic illustration of the antibacterial mechanism of Zn^2+^ ion.

The antibacterial efficiency of ZIF-8 depends on its concentration, which was firstly evaluated against *S. aureus*. With the increased concentration of ZIF-8 NPs, the antibacterial activity increases, and 100% of bacteria can be killed when the concentration reaches 160 μg/mL (Figure 2a). Zn^2+^ ions can interact directly with the negatively charged bacterial cell surface, leading to membrane disruption, a loss of structural integrity and the increased intracellular ROS accumulation [11,27]. Therefore, the intracellular ROS levels of *S. aureus* induced by different concentrations of ZIF-8 NPs were determined using a ROS assay kit. The fluorescence intensity of ROS is positively correlated with the concentration of ZIF-8 NPs (as shown in Figure 2b,c), indicating that higher concentrations of ZIF-8 NPs tend to produce higher levels of ROS to more effectively kill bacteria. Then, the antibacterial efficiencies of ZIF-8 NPs at 160 μg/mL against *E. coli* and MRSA were examined, and they were both over 99.9%, indicating the broad-spectrum antibacterial ability of ZIF-8. The status of bacteria for the 160 μg/mL group was further confirmed via Live/Dead staining (Figure 2d), and the groups without ZIF-8 treatment (labeled as Blank) were set as controls. After 1 day of culture, a mass of alive bacteria stained in green were observed in Blank groups, indicating their good health status, while most bacteria incubated with 160 μg/mL of ZIF-8 NPs were stained in red, indicating their death. SEM images also show that bacteria are plump and intact in Blank groups; however, nanoparticle-treated bacteria are clearly corrugated and even broken, further demonstrating their poor health status (Figure 2e). Zn^2+^ ions can interfere with essential enzymatic functions by binding to thiol groups of key metabolic proteins, thereby inducing the generation of ROS to inhibit bacterial respiration and DNA replication, membrane damage and subsequent bacteria death [10,20], as illustrated in Figure 2f. This bacterial kill mechanism makes Zn^2+^ a potent and broad-spectrum antibacterial agent.

### 3.2. ZIF-8-Containing Spay and Antibacterial Properties

**Figure 3:**
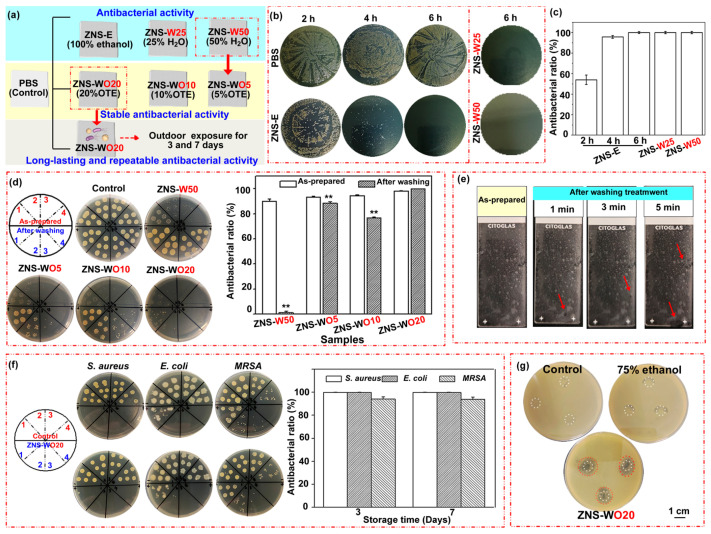
(**a**) A schematic illustration of the experimental groups and preparation process of different ZIF-8-based sprays, (**b**,**c**) the time-dependent antibacterial rates of ZNS-E, ZNS-W25, and ZNS-W50 against *S. aureus* after 2, 4, and 6 h of incubation, (**d**) the antibacterial performance of ZNS-W50, ZNS-WO5, ZNS-WO10, and ZNS-WO20 groups before and after five cycles of washing with detergent solution, (**e**) photographs of ZNS-WO20-coated glass slides after magnetic stirring in pure water for 0, 1, 3, and 5 min, (**f**) the antibacterial performance of infected ZNS-WO20 after 3 days and 7 days of outdoor placing, and (**g**) the zone of inhibition test against *S. aureus*. Numbers 1–4 marked in (**d**,**f**) indicate the corresponding dilution numbers of the bacterial suspension **: *p* < 0.01, compared with the As-prepared group.

For the preparation of ZNS spray, ZIF-8 NPs were dispersed in ethanol containing varying amounts of water and OTES. The mixture was then transferred into a commercial spray bottle and shaken well before use. Each spray application delivered approximately 160 μL of solution. Based on the antibacterial activity of ZIF-8 at different concentrations (Figure 2), the ZIF-8 concentration in the spray was set to 1 mg/mL. A total of 20 μL of bacterial suspension (~10^8^ CFU/mL) was firstly applied on each gauze slice (approximately 6 mm in diameter) and left to dry aseptically in a laminar flow cabinet at room temperature for 10 min. Then, a single spray was applied onto a gauze slice and incubated for different durations to evaluate the antibacterial performance. The experimental groups were established and named as illustrated in Figure 3a.

ZIF-8 NPs were first dispersed in absolute ethanol to form the spray (ZNS-E), and their antibacterial performance with the prolonged incubation time was examined with PBS as the control, as shown in Figure 3b. After incubation for 2 h, the sprayed gauze could kill about 55% of *S. aureus*, and with the incubation period prolonged to 4 h, more than 95% of bacteria were killed. When the incubation time was extended to 6 h, the antibacterial rate reached 100%. High ethanol concentrations may cause irritation to humans. Therefore, different proportions of water (25% and 50%) were added to replace part of the ethanol, forming sprays (named ZNS-W25 and ZNS-W50, respectively). Even when the ethanol concentration was reduced to 50%, the antibacterial efficacy still remained at 100% after 6 h of incubation (Figure 3b,c), indicating that this reduction does not affect the antibacterial performance of the spray. Sprays with much higher water ratios (e.g., 75% and 90%) were also tested; however, more water leads to longer gauze drying times. Considering application convenience, ZNS-W50 was selected to continue the following study.

OTES has a hydrolyzable triethoxysilyl anchoring group and a hydrophobic linear octyl chain. More OTES is beneficial for nanoparticles dispersing in aqueous solution and forming a uniform film on material surfaces [15]. Whether it affects the antibacterial performance of the spray is unknown. So, different proportions of OTES were added in ZNS-W50, named as ZNS-WO5, ZNS-WO10 and ZNS-WO20 according to the volume percent of OTES (Figure 3a). The antibacterial performance and stability of the obtained gauzes were then evaluated. Gauzes sprayed with pure PBS were used as controls. Part of the gauzes were further immersed in a 2 vol% commercial laundry detergent solution, stirred with a magnetic stirrer at 20 rps for 5 min, and then air-dried for 30 min; this process was repeated five times. The washed gauzes were applied to examine the antibacterial duration of the spray. The as-prepared ZNS samples could kill almost all the bacteria, and after washing treatment, the group without OTES (ZNS-W50) lost its antibacterial activity completely, which was due to the desorption of ZIF-8 during washing. When the OTES increased, the antibacterial activity increased, and more than 95% of bacteria were killed in the ZNS-WO20 group, indicating the maintenance of excellent antibacterial stability even after continuous regular washing. OTES acts as a dispersant, encapsulating the ZIF-8 nanoparticles and causing them to repel each other and disperse uniformly, and it also serves as a binder, adhering and immobilizing the ZIF-8 nanoparticles onto the surfaces of gauzes, increasing the anti-washing property [15,29]. The effect of OTES on the adhering stability was further examined on a glass slide, as shown in Figure 3e. The ZNS-WO20 spray formed uniform coatings on glass slides, which were further placed in pure water under magnetic stirring for different durations. As treatment time increased, the film retained its form well with some slight detachments (marked with arrows in Figure 3e), indicating the good adhesion and stability of the film on smooth surfaces.

Based on the stability and anti-washing results, ZNS-WO20 was selected to evaluate the long-lasting antibacterial performance of the sprayed gauze slices against *S. aureus*, *E. coli*, and MRSA. PBS-sprayed gauze slices were used as controls. First, 20 mL of bacterial suspension (~10^8^) was applied to the gauzes and air-dried for 30 min. The sprays were then applied to the inoculated gauzes, and the gauzes were placed outdoors for 3 and 7 days to further assess their antibacterial performance after outdoor exposure (Figure 3a). The gauzes still exhibited outstanding antibacterial activity against different types of bacteria even after 7 days of exposure, indicating long-lasting and repeatable antibacterial activity (Figure 3f).

Finally, the antibacterial performance of the spray was evaluated using the zone of inhibition test with PBS and medical 75% alcohol as comparisons. A bacterial suspension of *S. aureus* was spread evenly onto an agar plate and allowed to dry. Then, PBS, medical 75% alcohol, and ZNS-WO20-sprayed gauzes were applied onto the agar plate, which was subsequently incubated overnight in a bacterial incubator. The results are shown in Figure 3g. The medical alcohol group had no inhibition zone due to the rapid volatilization of ethanol from the gauze. The ZNS-WO20 group had an obvious inhibition zone, indicating the releasing antibacterial effect of the ZNS-WO20 gauze.

### 3.3. In Vitro Biocompatibility Testing of ZNS-WO20 Spray

Antibacterial spray products involve direct contact with the human body, necessitating biocompatibility testing to confirm their safety for human use. Biocompatibility testing evaluates a product’s toxicity and irritation potential to the human body, as well as its irritation and damage levels to skin. If a spray product fails to meet biocompatibility standards, it may cause adverse reactions like skin allergies, redness, swelling, itching, or burning pain, and could even lead to tissue damage. Therefore, testing biocompatibility is a crucial step in ensuring the safety and efficacy of antimicrobial spray products.

**Figure 4:**
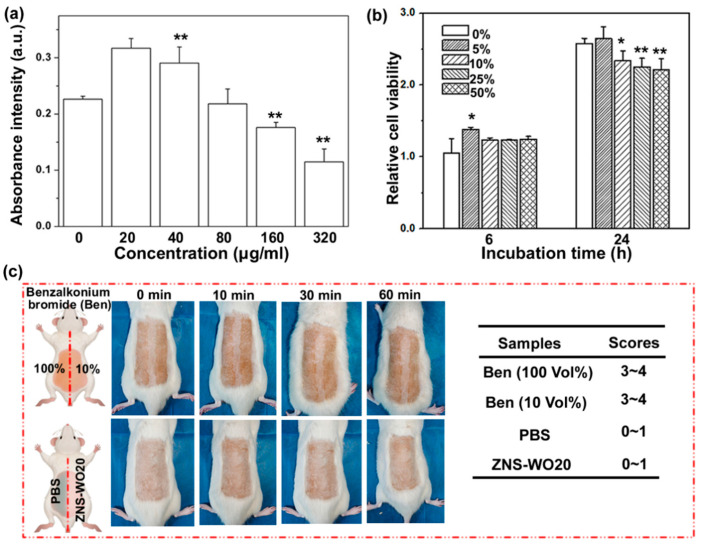
(**a**) The viability of L929 after 24 h of incubation with different concentrations of ZIF-8 NPs, (**b**) the viability of L929 cells cultured with serial dilutions of extracts from the ZNS-WO20-sprayed gauze, (**c**) an evaluation of dermal irritation in rats: photographs of rat dorsal skin after exposure to different sprays and the skin irritation scores. * *p* < 0.05 and ** *p* < 0.01, compared with the 0 μg/mL or 0% group.

Firstly, the biocompatibility of ZIF-8 NPs was evaluated at different concentrations, as shown in Figure 4a. With the increased concentration of ZIF-8, the cell viability increased initially and then decreased. At a concentration of 160 μg/mL, which can kill bacteria effectively, the cell viability was about 80% of that of the group without ZIF-8, indicating the biocompatibility of ZIF-8 NPs at 160 μg/mL. Zinc ions (Zn^2+^) serve as essential catalytic and structural cofactors for numerous enzymes, including DNA polymerases and superoxide dismutase [10]. At low doses, Zn^2+^ enhances cell proliferation, migration, and metabolic function by stabilizing protein folding and modulating signaling pathways such as MAPK and PI3K/Akt; however, excessive Zn^2+^ induces oxidative stress, mitochondrial dysfunction, and apoptosis, severely reducing cytocompatibility [12,30].

Then, ZNS-WO20 gauzes sprayed with ZIF-8 nanoparticles were immersed in culture medium to extract their solution. The extract mainly contained Zn^2+^ ions and imidazolate anions, which both have concentration-dependent biocompatibility [11]. For instance, moderate levels of Zn^2+^ (e.g., ~0.55 μg/mL) can activate the TGF-β1 signaling pathway to promote fibroblast proliferation and collagen synthesis, whereas excessive extracellular Zn^2+^ ions (e.g., ~5.2 μg/mL) induce ROS and subsequent cytotoxicity [9,25]. Therefore, the viability of L929 fibroblasts in diluted extracts was tested, with the results shown in Figure 4b. Cells maintained good viability across different concentrations of the extract. Notably, the conditioned medium containing 5% extract accelerated cell adhesion to some extent compared with the complete medium (0% extract). As the extract concentration increased, cell viability showed no obvious change at 6 h of incubation and decreased slightly at 24 h of incubation, but remained above 70% of the viability observed in the complete medium. These results indicate the good biocompatibility of the ZNS-WO20 gauze. Finally, the skin irritation potential of ZNS-WO20 spray was assessed, with PBS and benzalkonium bromide (Ben) as the negative and positive control, respectively (Figure 4c). After depilation of rats, samples were sprayed to their backs to observe skin reactions. The negative control (PBS) shows no changes on rat skin surfaces. The positive control (Ben) exhibits noticeable redness and allergic reactions on backs, even when Ben was diluted into 10 vol%. In contrast, the backs treated with ZNS-WO20 spray showed no significant changes, similar to the negative control groups. The skin irritation scorings of different groups are shown in the table in Figure 3c. All these results demonstrate that ZNS-WO20 spray is biocompatible and friendly to skin.

## 4. Conclusions

Rhombic dodecahedral ZIF-8 NPs with an average size of 245 nm were successfully synthesized via a solvothermal method. ZIF-8 NPs exhibited concentration-dependent and broad-spectrum antibacterial activity, achieving nearly 100% inhibition against *S. aureus*, *E. coli*, and MRSA at 160 μg/mL. Mechanistically, ZIF-8 responsively releases Zn^2+^ ions in acidic environments, generating abundant intracellular ROS that leads to bacterial death. Furthermore, an antibacterial spray composed of ZIF-8 with 50% ethanol and 20% OTES was formed. OTES acts as both a dispersant and a binder, significantly improving the adhesion and anti-washing durability of the spray on various substrates, including gauze and glass. The sprayed coatings retain >95% antibacterial efficacy even after repeated washing and seven days of outdoor exposure. The ZNS-WO20 spray exhibits excellent biocompatibility and no skin irritation, highlighting its safety for human contact.

This work integrates a pH-responsive MOF with a silane-based spray system to achieve long-lasting, wash-resistant antibacterial performance and biocompatibility, simultaneously. This ZIF-8 spray holds great promise for easy preparation, economy and diverse applications, including environmental sanitation, and personal protection to mitigate pathogen spread in daily life and clinical settings.

## Figures and Tables

**Table 1:** Skin irritation scoring criteria.

Reaction Erythema and Eschar Formation	Score
No erythema	0
Very slight erythema (barely perceptible)	1
Well-defined erythema	2
Moderate to severe erythema	3
Severe erythema (beet-redness) to eschar formation	4

## Data Availability

The original contributions presented in this study are included in the article. Further inquiries can be directed to the corresponding authors.
